# Transcription factor-driven regulation of ILC1 and ILC3

**DOI:** 10.1016/j.it.2022.04.009

**Published:** 2022-07

**Authors:** Jan-Hendrik Schroeder, Jane K. Howard, Graham M. Lord

**Affiliations:** 1 School of Immunology and Microbial Sciences, King’s College London, London, UK; 2 School of Cardiovascular and Metabolic Medicine & Sciences, King’s College London, London, UK; 3 School of Biological Sciences, Faculty of Biology, Medicine and Health, Division of Infection, Immunity and Respiratory Medicine, University of Manchester, Manchester, UK

**Keywords:** innate lymphoid cells, transcription factor

## Abstract

Mammalian innate lymphoid cells (ILCs) have functional relevance under both homeostatic and disease settings, such as inflammatory bowel disease (IBD), particularly in the context of maintaining the integrity of mucosal surfaces. Early reports highlighted group 1 and 3 ILC regulatory transcription factors (TFs), T-box expressed in T cells (T-bet; *Tbx21*) and RAR-related orphan nuclear receptor γt (RORγt; *Rorc*), as key regulators of ILC biology. Since then, other canonical TFs have been shown to have a role in the development and function of ILC subsets. In this review, we focus on recent insights into the balance between mature ILC1 and ILC3 based on these TFs and how they interact with other key cell-intrinsic molecular pathways. We outline how this TF interplay might be explored to identify novel candidate therapeutic avenues for human diseases.

## Highlights

ILCs have an important role in mammalian tissues, providing an early immune response and a regulatory function to modify the adaptive and epithelial immune response.
Group 1 ILCs (ILC1) are heterogenous depending on the role of either T-bet or Hobit as TFs for maintenance.
Group 3 ILC (ILC3) express several TFs that appear to inhibit their plasticity toward ILC1, and these include RORγt, RORα, c-Maf, and HIF1. By contrast, T-bet, in conjunction with Aiolos and Bcl6, promote ILC1.
The balance between ILC3 and ILC1 appears to be crucial for the severity outcome of IBD, colorectal cancer, and likely other diseases.

## Clinical relevance of ILC1 and ILC3

ILCs are particularly abundant in mammalian mucosal tissues, where they elicit an early immune response and interact with CD4^+^ T cells and the epithelium [1., 2., 3., 4., 5., 6., 7., 8., 9.]. ILC subsets are distinct from natural killer (NK) cells and have been categorized into four groups, depending on the characteristic expression of TFs defining **T-bet**^+^ (see Glossary) type 1 ILC (ILC1), **GATA-binding protein 3** (GATA3)^+^ type 2 ILC (ILC2), **RORγt**^+^ type 3 ILC (ILC3), and RORγt^+^ lymphoid tissue inducer cells (LTi) ILC3 in mice and humans [2,3] (Box 1). This review focuses on the roles of ILC1 and ILC3 because the balance between these has been associated with inflammatory bowel disease (IBD) and colorectal carcinoma (CRC) [2,3,8].

Box 1 **Identification of ILCs**

Literature on ILCs is often compounded by the lack of ILC-specific markers, a non-uniform use of lineage markers to define non-ILC leukocytes in the gating strategy, or by diverse gating strategies to analyze ILC1 and ILC3 subsets. In terms of lineage markers, the use of CD3 and a B cell marker, such as CD19, is the minimal requirement for detecting ILCs in mouse and human, but it is recommended and practiced in most studies reviewed here to use additional lineage markers targeting macrophages, neutrophils, granulocytes, and platelets. Some studies also use CD5 in the lineage cocktail [10., 11., 12., 13., 14., 15., 16., 17., 18., 19., 20., 21., 22., 23.]. However, the use of CD5 as a lineage marker is controversial because ILCs have been reported to express CD5, while CD5^+^ ILCs have also been shown to express αβTCR and have been related to nonfunctional T cells [2,24,25]. Furthermore, CD127 is often used as a mouse and human ILC marker; however, CD127 expression in murine ILCs can be lost upon stimulation with IL7 [18., 19., 20.,26., 27., 28., 29., 30., 31., 32., 33., 34., 35.]. Similarly, CD90 expression, in particular its high expression, has been used in a few studies to detect ILC3 [12,14,22,36., 37., 38., 39., 40.]. However, preliminary data suggest that CD90 expression in ILCs is also lost *in vitro* and *in vivo*, and that ILC1 and ILC2 might have high expression of CD90 [18] (pers. commun. from J.H.S. and G.M.L.), although this remains to be robustly validated. CD49a is sometimes used as a murine ILC1 marker in studies investigating ILC1 from non-intestinal tissues [11,14,16,41., 42., 43., 44., 45., 46.]. The detection strategies in other mouse studies reviewed here consider the fact that ILC1 do not express RORγt, while it is a key marker of ILC3.

Alt-text: Box 1

ILC1 have a beneficial role during the early immune response in mice to Mouse Cytomegalovirus (MCMV) in the liver, to *Toxoplasma gondii* in the brain, and to epithelial infection with *Cryptosporidium parvum*, but can also be pathogenic in association with colitis in mouse models, and during IBD in humans [1,19,28,29,47,48]. The detrimental effect of ILC1 appears to be mediated by the production of IFNγ and TNFα, both of which induce apoptosis of epithelial cells *in vitro*, which is likely to contribute to disruption of the intestinal barrier *in vivo* and to microbiota-driven inflammation [9,49., 50., 51.]. Beyond inflammation, the presence of ILC1 has been associated with a detrimental role in human CRC and in several murine tumor models, such as azoxymethane (AOM)/**dextran sulfate sodium (DSS)**-induced CRC, the B16 lung metastasis model, and subcutaneous injection of MCA-induced primary tumor lines [8,41,52]. Moreover, ILC1 produce TGFβ, inducing a cancer-associated transcriptional profile in intestinal organoids *in vitro* [9]. In addition, ILC1 have been attributed a poor ability to mount antitumor responses due to their limited cytotoxicity ability [8,41]. Furthermore, hepatic ILC1 express IL2R, which restricts the access of CD8^+^ T cells to IL2 *in vitro*, and also in the tumor environment (TME), although this remains to be rigorously demonstrated *in vivo* [53].

ILC3 are less frequent in the murine liver than are ILC1, but they have a broadly protective role in the gut unlike ILC1, based on findings from several mouse models of *Citrobacter rodentium* infection- or DSS-elicited colitis, as reviewed recently [53,54]. The protective role of ILC3 during colitis is founded on their ability to express IL2, MHC class II (MHCII), and OX40L, which is relevant for their effect on T cell responses [4., 5., 6.,55., 56., 57.]. Specifically, ILC3-derived IL2 and the direct cell interaction via OX40L:OX40 both promote the abundance of **regulatory T cells (Tregs)** in the mouse gut [5,6]. In the absence of IL2 expression in *Rag1*^–/–^ × *Rorc-Cre* × *Il2*^fl/fl^ mice, more severe colitis is induced upon adoptive transfer of naïve CD4^+^ T cells, compared with *Rag1*^–/–^ × *Il2*^fl/fl^ mice, demonstrating the relevance of ILC3-derived IL2 in modulating pathogenic T cells [5]. Moreover, CD80 or CD86 expression is barely detectable in intestinal and **mesenteric lymph nodes** **(MLNs)** and intestinal **lamina propria** **(LP)** MHCII^+^ ILC3, which might be a main reason why these cells are dysfunctional in activating T cells *in vitro* and *in vivo* [7,55,56]. Of note, *Rorc* is expressed only by T cells and ILCs, and mouse T cells do not express MHCII; this allows the selective genetic deletion of MHCII in RORγt^+^ ILCs in the presence of an intact adaptive immune system, as generated in *Rorc-Cre* × *H2-Ab1*^fl/fl^ mice [55]. Thus, in mouse MLN, MHCII^+^ ILC3 negatively regulated **follicular T helper cells** **(T**_**FH**_**)**, causing reduced IgA production and preventing the development of spontaneous colitis in *H2-Ab1*^fl/fl^ mice (littermate controls) relative to *Rorc-Cre* × *H2-Ab1*^fl/fl^ mice [4,55,56]. This demonstrated that MHCII^+^ ILC3 could restrain T_FH_-driven immune responses to avoid inflammation at the intestinal barrier in this scenario. However, MHCII^+^ ILC3 can also promote a pathogenic T cell response during neuroinflammation because *H2-Ab1*^fl/fl^ mice develop a worse outcome in the murine experimental autoimmune encephalomyelitis (EAE) model of multiple sclerosis, with greater T cell activation relative to *Rorc-Cre* × *H2-Ab1*^fl/fl^ mice; this suggests that MHCII^+^ ILC3 also drives a pathogenic T cell response in other organs [58]. Similarly, in addition to promoting Tregs, ILC3 expressing OX40L (encoded by *Tnfsf4*) can promote a pathogenic Th1 response in the intestine upon adoptive transfer of T cells, and the disease outcome is ameliorated if the recipient mice harbor a *Rorc-Cre* × *Tnfsf4*^fl/fl^ background [57].

Furthermore, ILC3 also drive colitis in the presence of pathogenic *Helicobacter* spp. as part of the microbiota [59,60]. In line with these mouse models, intestinal ILC3 in patients with IBD exhibited reduced expression of MHCII and IL2 and greater OX40L expression compared with healthy control individuals, suggesting that ILC3 act as key modulators for this disease [5,56,57]. In contrast to intestinal ILC3, MHCII^+^ ILC3 in the spleen expressed CD80 and CD86 upon adoptive transfer of RORγt-fate mapper-marked *Rag2*^−/−^ × *Rorc*^fm+^ mouse splenic or small intestine (SI) ILC3 into *Rag2*^−/−^ × *Il2rg*^*−/−*^ mice that were deficient in mature ILC [7]. This indicates that CD80 and CD86 are dynamically expressed in ILC3 depending on the tissue they infiltrate; however, the stimulatory cues regulating their expression are unknown and do not appear to include IL23 and IFNγ; indeed, the expression of CD80 and CD86 in NKp46^–^ ILC3 remains unaltered in *Il23p19*^–/–^ and *Ifng*^–/–^ mice relative to wild-type mice [7,61]. These splenic ILC3 can drive T cell activation *in vitro.* However, it remains unknown whether intestinal ILC3 can migrate to the spleen to activate T cells [7,61]. Yet, in a model of CRC, MHCII^+^ ILC3 promoted Th1 and cytotoxic CD8^+^ T cell (CTL) responses in the AOM/DSS model of CRC, restricting cancer progression, as evidenced from the restricted differentiation of Th1 and CTL in *Rorc-Cre* × *H2-Ab1*^fl/fl^ mice relative to *H2-Ab1*^fl/fl^ control mice [8,62]. Therefore, it is tempting to speculate that, upon CRC formation, intestinal MHCII^+^ ILC3 might co-express CD80 and CD86 and activate T cells in secondary lymphoid organs.

Of note, the pathogenic role of ILC3 has also been reported to be driven by their production of GM-CSF, promoting colitis-driving ‘M1-like’ inflammatory macrophage differentiation. Specifically, upon antibody-mediated depletion of CD90^+^ ILC in colonic macrophages from DSS-treated mice (and relative to isotype control-treated DSS-treated mice), a reduced ‘M1-like’ RNA sequencing (RNA-seq) transcription signature was noted in macrophages; furthermore, a positive correlation of ILC and *CSF2* (encoding GM-CSF) single-cell (sc)RNA-seq gene expression with an ‘M1-like’ transcription signature in macrophages from intestinal biopsies of patients with IBD was reported [63,64]. Another study suggested that ILC3-derived IL17A is most likely an important mediator promoting neutrophilia, because greater ILC3 cellularity was correlated with enhanced neutrophilia in mouse intestine, which might have implications for inflammatory immune responses [20,65]. While IL17A may have a pathogenic role, it can also promote the maintenance of the epithelium in conjunction with IL22 and heparin-binding epidermal growth factor-like growth factor (HB-EGF); thus, we speculate that these three mediators are most relevant if the epithelium is exposed to apoptosis-stimulating TNFα and IFNγ because these cytokines might directly cause damage and disruption to the mucosal barrier [9,49., 50., 51.]. Moreover, intestinal ILC3-derived IL22 might promote barrier protection via enhanced proliferation and repair of the epithelium along with production of antimicrobial peptides because IL22 in general can elicit these effects; however, IL22 stimulation has also been reported to contribute to cancer development, as evidenced from several mouse CRC, pancreatic, hepatic, and other cancer models in which IL22 expression was positively correlated to tumor burden, as reviewed recently [54,66]. This has suggested an ambiguous role for IL22, in which it might provide a protective function in maintaining the mucosal barrier but might be detrimental by contributing to promote tumor growth, a seeming contradiction or ambivalence that warrants further investigation. In mouse models of liver inflammation, ILC3 were reported to drive a protective role, given that the presence of hepatic IL22^+^ ILC3 was associated with less histological liver damage upon induced hepatic ischemia reperfusion injury in *Rag*^–/–^ × *Rorc*^+/–^ compared with *Rag*^–/–^ × *Rorc*^–/–^ mice lacking ILC3 [67]. Although this might indicate a protective role of ILC3 in the liver, it cannot be excluded that ILC3 might also have a detrimental role in the liver in a context-dependent manner [67].

In contrast to ILC3, IL2 expression in ILC1 is barely detectable in mice, and MHCII expression has not been detected in human intestinal ILC1 [5,56]. This further supports the notion that ILC1 appear to contribute to driving inflammation and cancer development, as outlined in the preceding text. Hence, a balanced ILC3 and ILC1 response is required to restrain the ILC1-driven immune response promoting tissue damage, inflammation, and cancer. This balance appears to be mediated by the plasticity that has been documented whereby ILC1 differentiation can be achieved from an ILC3 precursor (ILC3–ILC1 plasticity) and vice versa [28,29]. This ILC1:ILC3 balance is well described in human and murine intestines [2,3]; indeed, a balance shift toward ILC1 differentiation was observed in patients with IBD and in mice developing colitis or CRC [8,19,28,29,68,69]. This highlights the importance of regulating the balance between pathogenic ILC1 and ILC3, which appear to have a predominantly protective role. In recent years, much progress has been made to understand the TF interplay regulating the balance between ILC1 and ILC3, as well as determining which are the relevant TFs that contribute to driving the maintenance and function of mature ILC1 and ILC3. This knowledge is vital for identifying ILC-specific putative therapeutic strategies that might modulate the ILC1:ILC3 balance, and which might, for instance, benefit patients with IBD or CRC. In this review, we discuss the emerging TF interplays that are essential for ILC1 and ILC3, as well as the balance between ILC1 and ILC3 in mice, and to some extent, in humans; we also outline how relevant TFs might relate to ILC function in CRC and/or IBD.

## Transcriptional control of murine ILC1 and ILC3 maintenance and functionality

### ILC1

#### T-bet-dependent ILC1

**Figure 1:**
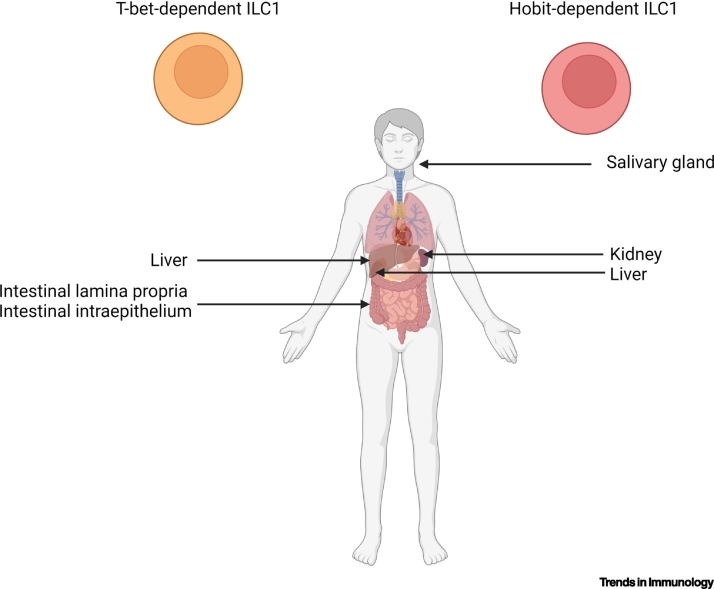
Group 1 innate lymphoid cell (ILC1) heterogeneity across tissues in mice. ILC1 are heterogenous depending on the key requirement for either T-box expressed in T cells (T-bet) or Homolog of Blimp-1 in T cells (Hobit) for development and maintenance. These ILC1 subsets are located in distinct anatomical sites. Hepatic granzyme C^+^ ILC1 require T-bet for maintenance; however, the role of Hobit in these cells remains to be verified. Figure created using BioRender (https://biorender.com).

SI LP ILC1 are absent in *Tbx21*^–/–^ mice and *Ncr1-Cre* × *Tbx21*^fl/fl^ mice and, overall, their development depends on TF T-bet [10,36,70., 71., 72., 73.] (Box 2 and Figure 1). Intestinal LP ILC1 are also almost absent in the colonic and SI LP if T-bet is temporally ablated in *Tbx21*^fl/fl^ × ***Cre-Ert2*** mice [19]. Moreover, NKp46 (encoded by *Ncr1*) and NK1.1 expression in these ILC1 is diminished shortly after induced depletion of T-bet in these mice, indicating that T-bet promotes their expression [19]. The key T-bet-dependency of intestinal LP ILC1 is distinctly different from NK cells or from intestinal **intraepithelial lymphocyte** **(IEL)** ILC1, residing within the intestinal epithelial barrier [19,69,74., 75., 76.] (Box 2). Moreover, colonic LP (cLP) ILC1 abundance is not crucially affected in *Stat1*^–/–^ or *Stat4*^–/–^ mice, as evidenced from their unaltered prevalence among ILC compared with wild-type ILC1; however, the number of ILC that are able to produce IFNγ in these mutant mice is reduced relative to wild-type mice [19,81]. This has suggested that STAT1 and STAT4 have a functional role in inducing T-bet expression in mature ILC1, but not during ILC1 development, although this remains to be further investigated [19,81].

Box 2 **Requirement for T-bet in NK, IEL ILC1 and LTi cells**

T-bet is important in promoting NK cell maturation, and mice deficient in *Tbx21* (encoding T-bet) and *Ncr1-Cre* × *Tbx21*^fl/fl^ mice have reduced cellularity of NK cells [10,74., 75., 76.]. In contrast to *Eomes*^–/–^ NK cells, splenic *Tbx21*^–/–^ NK cells are fully functional in terms of cytotoxicity (cytotoxicity assay using RMA-KR-derived NanoLuc cells as targets), but impaired in their capacity to produce IFNγ upon IL12 and IL18 stimulation *in vitro* [76,77]. Maintenance of colon and SI IEL ILC1, which do not express CD127, is also only partially dependent on T-bet postdevelopment, as determined in *Tbx21*^fl/fl^ × *Cre-Ert2* mice upon temporally induced depletion of T-bet [19]. In addition to these data, cellularity of SI IEL ILC1 is also reduced, but not lacking, in *Tbx21*^–/–^ mice [69]. Overall, these data indicate that T-bet is not centrally required for NK cell and IEL ILC1 development. Moreover, RNA-seq analyses revealed that NK cells transdifferentiate into intratumoral IEL ILC1-like cells in patients with head and neck cancer, suggesting that IEL ILC1 constitute NK cell progeny, although this has not been reported for intestinal LP ILC1 [78].

CCR6^+^ LTi are not believed to express T-bet and Aiolos, and their cellularity as well as RORγt and CD127 expression are not altered in the intestinal LP of *Tbx21*^–/–^ mice [13,20,79]. c-Maf also has no functional role in promoting RORγt, IL17A and IL22 expression in these cells or their cellularity in *Rorc-Cre* × *Maf*^fl/fl^ mice [34,80]. However, an uncharacterized NKp46^+^ CCR6^+^ ILC3 population appeared in both *Rorc-Cre* × *Maf*^fl/fl^ and *Il7r-Cre* × *Maf*^fl/fl^ mice, and CCR6^+^ ILC3 exhibited modest expression of T-bet in *Il7r-Cre* × *Maf*^fl/fl^ mice [34,80].

Alt-text: Box 2

#### Hobit-dependent ILC1

Hepatic ILC1 are detectable in *Ncr1-Cre* × *Tbx21*^fl/fl^ mice, indicating that hepatic and intestinal LP ILC1 differ in their requirement for T-bet [10]. However, the hepatic ILC1 population is significantly, but not entirely, diminished in **Homolog of Blimp-1 in T cells** **(Hobit)**-deficient (*Zfp683*^–/–^) and *Ncr1-Cre* × *Zfp683*^fl/fl^ mice, suggesting that the TF Hobit has a more central role for a subset of ILC1 than was previously thought [1,11,14,43,44]. Moreover, low CD127, but enhanced expression of CD3ε and granzyme B in a subset of hepatic ILC1 (which are ablated in *Zfp683*^–/–^ mice) demonstrated a characteristic difference between Hobit-dependent and conventional ILC1, which might prove to be relevant in future investigations [14] (Figure 1). These CD127^-^ ILC1 also express less T-bet and IFNγ compared with CD127^+^ ILC1 [14]. However, Hobit-dependent ILC1 exhibit a transcriptional profile that is closely related to ILC1 and might be generated from a hepatic CD127^+^ ILC1 progenitor, as evidenced from the adoptive transfer of congenically marked hepatic CD127^+^ ILC1 into *Rag2*^–/–^ × *γc*^–/–^ mice, or in CD127^+^ ILC1 cell cultures with **OP9-DL1** in the presence of IL2 and IL15, which both resulted in granzyme B and granzyme A expression in these cells [14]. Furthermore, induced ablation of Hobit in *Zfp683*^fl/fl^ × *Cre-Ert2* mice resulted in lower granzyme B and enhanced CD127 expression in hepatic ILC1, suggesting a postdevelopmental role of Hobit in this ILC1 subset because Hobit ablation was induced in adult mice [14]. Of note, Hobit expression in ILC1 has also been detected in salivary gland, kidney, and MLN, while its expression is lower in SI LP and IEL ILC1 in mice [14,41,44].

At least some of the hepatic and salivary gland Hobit^+^ ILC1 derive from an NK cell precursor, and this plasticity is inhibited by the TGFβ signaling inhibitor Smad4, as demonstrated by the greater abundance of ILC1 in *Ncr1-Cre* × *Smad4*^fl/fl^ mice compared with controls [14,41,44]. These hepatic ILC1 are less cytotoxic than NK cells, but the expression of Hobit is induced in hepatic NK cells upon MCMV infection, suggesting that NK cell-derived ILC1 emerge upon infection [14,41,44,82]. Furthermore, in *Ncr1-Cre* × *Tgfbr2*^fl/fl^ mice, the abundance of salivary gland ILC1 is reduced compared with *Ncr1-Cre* × *Tgfbr2*^wt/fl^ mice [16]. Hence, these studies suggest that Hobit-dependent ILC1 reside in the liver and salivary gland and may develop from Hobit-independent ILC1 or NK cells via TGFβ signaling [14,16,41,44,82]. Of note, lineage clustering of **assay for transposase-accessible chromatin using sequencing** **(ATAC-seq)** similarity revealed a close similarity between liver ILC1 and NK, suggesting a close relationship between these two cell types and that hepatic ILC1 may derive from NK cell precursors [83]. In contrast to NK cells, a subset of hepatic and salivary gland ILC1 express granzyme C (encoded by *Gzmc*), but co-express granzyme B and perforin, similarly to NK cells [46]. These granzyme C^+^ ILC1 are almost ablated in the liver and are less frequent in the salivary gland in *Gzmc-Cre* × *Tbx21*^fl/fl^ mice compared with wild-type mice [46]. Similarly, granzyme C^+^ cells are also detected in a subcluster of SI LP ILC1/**ex-ILC3** by scRNA-seq, and this subcluster is absent in *Id2-Cre-Ert2* × *Rorc*^fl/fl^ × *Tbx21*^fl/fl^ mice upon T-bet ablation, suggesting that granzyme C^+^ ILC1 in the intestine require T-bet for maintenance [13]. Moreover, **trajectory analyses** of these data indicate that granzyme C^-^ and granzyme C^+^ ILC1/ex-ILC3 originate from distinct developmental pathways [13]. However, the relationship between these Granzyme C^+^ ILC1 and NK cells is yet to be determined. Partial Eomes expression was detected in granzyme C^+^ ILC1 in the salivary gland and tumors from a PyMT breast cancer mouse model, but not in the liver, indicating that granzyme C^+^ ILC1 and NK cells may be closely related [46]. It is unknown whether granzyme C^+^ ILC1 express Hobit or how they relate to Hobit-dependent ILC1, but granzyme C^+^ ILC1 also appear to lack the capacity to express IFNγ, at least at steady state, in a way that is similar to Hobit-dependent ILC1 [14,46]. Of note, hepatic ILC1 do not require signaling through the TGFβ receptor, but salivary gland granzyme C^+^ ILC1 are partially ablated in *Gzmc-Cre* × *Tgfbr2*^fl/fl^ mice [46]. Lastly, hepatic ILC1 are diminished in *Ncr1-Cre* × *Ifng*^fl/fl^ compared with wild-type mice, indicating that IFNγ derived from undefined NKp46^+^ hepatic lymphocytes might be functionally relevant in promoting the *in vivo* generation of hepatic ILC1 [14,77,84]. Whether IFNγ, a T-bet target, is also of relevance for granzyme C^+^ ILC1 remains unknown.

Overall, novel insight reveals that ILC1 are distinct from NK cells and comprise distinct subsets dependent on the key requirement of either T-bet or Hobit. It is unknown how these ILC1 subsets relate to T-bet-dependent granzyme C^+^ ILC1 because the role of Hobit in these cells is yet to be determined.

### ILC3

Although ILC3 are absent in mice deficient in *Rorc* (encoding RORγt), RORγt is not crucial for the postdevelopmental maintenance of intestinal LP ILC3 [13,70,73,85]. All SI LP ILC3 subsets [CCR6^+^ LTi, NKp46^+^ ILC3m and NKp46^–^ CCR6^–^ ‘double negative’ (DN) ILC3] are preserved upon induced depletion of RORγt in *Id2-Cre-Ert2* × *Rorc*^fl/fl^ adult mice [13,85]. These SI LP ILC3 subsets exhibit a preserved capacity to produce IL22 and IL17A in these mice, but the cellularity of IL17^+^, but not IL22^+^, NKp46^+^ ILC3, and DN ILC3, is reduced relative to *Id2-Cre-Ert2* mice [13]. By contrast, SI LP CCR6^+^ LTi production of IL17A and IL22 has been reported to be independent of the postdevelopmental expression of RORγt in these mice, indicating that other TFs may have a role [13].

**Retinoic acid receptor-related orphan receptor α (RORα)** may also be required for ILC3 development or maintenance. Approximately 55% of SI ILC3 in adult mice have expressed this TF in the past, as determined using a fate-mapper mouse model [86]. Furthermore, congenically marked cecal ILC3 in recipient wild-type mice developed at a lower frequency if the adoptively transferred bone marrow cells were derived from ‘staggerer’ (sg) mice with a loss-of-function mutation in *Rora*, indicating a role for RORα in ILC3 development or maintenance [35,86,87]. Induced temporal co-ablation of RORγt and RORα expression in *Id2-Cre-Ert2* × *Rorc*^fl/fl^ × *Rora*^fl/fl^ mice indicated that, upon induced depletion of RORγt, RORα was important in maintaining a substantial degree of IL17A expression in SI LP CCR6^+^ LTi, DN ILC3, and eventually NKp46^+^ ILC3, in *Id2-Cre-Ert2* × *Rorc*^fl/fl^ mice [13]. Studies with this experimental design have also demonstrated that mature SI LP NKp46^+^ ILC3 require RORα for IL22 production upon ablation of RORγt, but this was not observed in CCR6^+^ LTi and DN ILC3 [13]. Overall, RORγt and RORα appear to have a functional role in mature ILC3 for the production of cytokines, but not for their maintenance.

When examining the role of TF GATA3, depletion of GATA3 in *Vav-Cre* × *Gata3*^fl/fl^ mice resulted in loss of SI LP NKp46^+^ and DN ILC3, and diminished SI LP CCR6^+^ LTi and ILC1, relative to *Gata3*^fl/fl^ mice [13,21,22,42,79,86]. Similarly, in *Rorc-Cre* × *Gata3*^fl/fl^ mice, the diminished cellularity of all SI LP ILC3 subsets and the presence of ILC3-derived ILC1 were observed without affecting the overall cellularity of ILC1 compared with *Gata3*^fl/fl^ mice [79]. However, ILC1 were ablated in at least the liver and brain of *Ncr1-Cre* × *Gata3*^fl/fl^ mice, indicating that GATA3 might also be essential for ILC1 development [48].

Intestinal LP ILC3 maintain low expression of GATA3 binding in the *Rorc* and *Il7r* loci [13,22,79]. This interaction leads to enhanced expression of RORγt and CD127 in SI LP and cLP ILC3 and greater cellularity of SI LP ILC3 [79]. GATA3 has also a substantial impact on the genetic profile of SI LP ILC3 [79]. For instance, IL22 and Arginase-1 are induced by GATA3 at the transcriptional level in SI LP NKp46^+^ ILC3 and CCR6^+^ LTi, as determined from *Rorc-Cre* × *Gata3*^fl/fl^ mice [79]. Arginase-1 expression in mature SI LP ILC3 is also promoted by RORγt and RORα, as evidenced by its reduced expression in *Id2-Cre-Ert2* × *Rorc*^fl/fl^ × *Rora*^fl/fl^ mice or *Id2-Cre-Ert2* × *Rorc*^fl/fl^ compared with *Id2-Cre-Ert2* mice [13]. It remains to be determined whether there is a common pathway for these three TFs and for the role of arginase-1 in ILC3 [13]. Of note, GATA3 can also control IL22 expression in cLP ILC3, while IL17A expression remains unaffected, as evidenced from multiplex cytokine assay analysis using supernatants from cLP leukocytes from *C. rodentium*-infected *Rag1*^–/–^ × *Rorc-Cre* × *Gata3*^fl/fl^ and *Rag1*^–/–^ × *Gata3*^fl/fl^ mice [79]. This might indicate that IL22 expression in ILC3 is controlled, at least partially, by a GATA3-dependent pathway, which has no role in regulating IL17A production in these cells.

GATA3 can also promote **Aryl hydrocarbon receptor** **(AHR)** expression in SI LP NKp46^+^ ILC3 and CCR6^+^ LTi [79]. This is relevant because AHR has an important role at barrier tissues, including the gut, and is activated by several ligands of dietary, microbial, or environmental origin [88]. AHR protein expression can be detected in SI LP and cLP T-bet^-^ and T-bet^+^ ILC3, and its overexpression in *Ahr*^*dCAIR/+*^ mice results in greater cellularity of cLP and SI LP ILC3 [15]. Conversely, *Rorc-Cre* × *Ahr*^fl/fl^ or *Ahr*^–/–^ mice have significantly diminished cellularity of all LP ILC3 subsets in the SI, colon, and cecum compared with wild-type mice [36,38., 39., 40.,^89^]. In addition, residual ILC3 in *Ahr*^–/–^ colon and SI NKp46^+^ and NKp46^-^ ILC3 show impaired production of IL22, while IL17A expression remains unaffected [89].This implicates AHR as a possible regulator of IL22 and that it may act in this function downstream of GATA3.

Intestinal LP and hepatic ILC1 also express AHR, and the development of hepatic ILC1 is partially dependent on this TF, as evidenced from the defective development of these cells from adoptively transferred congenically marked *Ahr*^–/–^ fetal hepatic cells [13,15,30,45]. The role of GATA3 in mature ILC1 is yet to be determined, but, given the role of AHR in ILC1 and the apparent role of GATA3 in ILC1 development, we speculate that a GATA3–AHR axis might be relevant for both ILC3 and ILC1.

RORγt, RORα, GATA3, and AHR have a crucial role in ILC3, and their cellularity is significantly diminished in mice deficient of their respective TFs; however, in mature ILC3, RORγt and RORα are not necessary for their maintenance, but are for their functionality, such as in IL17A production. The GATA3–AHR axis appears to drive ILC3 cellularity and IL22 production, but its exact role in mature ILC3 remains undefined. ILC1 cellularity may also depend on GATA3 and AHR, and we speculate that GATA3–AHR is functional in ex-ILC3 ILC1.

### ILC1-ILC3 balance

The TF interplay controlling the ILC1:ILC3 balance is complex and involves many TFs in addition to T-bet and RORγt. Relevant studies have often used mouse models in which the TF of interest is depleted under the control of a Cre-linked gene that may be already expressed in ILC progenitors, such as IL7R, due to the lack of knowledge of genes specifically expressed in mature ILC subsets [73]. This complicates obtaining a definitive statement on whether the shifts in the ILC1:ILC3 balance are driven by postdevelopmental plasticity or are due to developmental processes (see Outstanding questions).

#### T-bet and RORγt counterbalance

**Figure 2:**
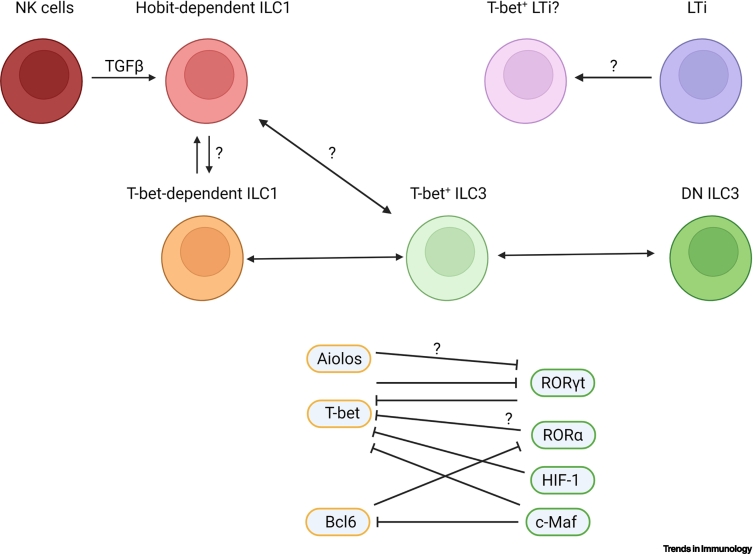
Key figure. Model of transcription factor (TF) interplay regulating Group 3 innate lymphoid cell (ILC3)–Group 1 innate lymphoid cell (ILC1) plasticity in mice and humans. TGFβ crucially regulates natural killer (NK) cell plasticity toward Hobit-expressing ILC1, which may present Hobit-dependent ILC1. The relationship of Homolog of Blimp-1 in T cells (Hobit)- and T-box expressed in T cells (T-bet)-dependent ILC1 remains unknown, as does whether plasticity exists between these ILC1 subsets. Plasticity between T-bet-dependent ILC1 and ILC3 is regulated by a TF interplay, as depicted. T-bet and RAR-related orphan nuclear receptor γt (RORγt) regulate each other, and c-musculoaponeurotic fibrosarcoma(c-Maf) and hypoxia-inducible factor (HIF) 1 can directly inhibit T-bet expression. Whether T-bet is also directly regulated by B cell lymphoma 6 (BCL6)-inhibited RORα and whether Aiolos and the c-Maf-controlled BCL6 inhibit RORγt remains to be determined. RORγt^+^ lymphoid tissue inducer cells (LTi) ILC3 are not believed to express T-bet; however, T-bet-expressing LTi ILC3 have been observed in c-Maf-deficient mice. Figure created using BioRender (https://biorender.com). Abbreviation: DN, double negative.

ILC3–ILC1 plasticity describes the transformation of DN ILC3 to T-bet-expressing NKp46^+^ ILC3 and subsequently to ILC1 [13,36] (Figure 2, Key figure). The counter-regulation between RORγt and T-bet controls the ILC3:ILC1 balance, as demonstrated by the reduced frequency of NKp46^+^ ILC3 and enhanced expression of RORγt in cLP DN ILC3 in *Tbx21*^–/–^ mice [13,20,36]. ILC3–ILC1 plasticity occurs in mature ILCs, given that induced depletion of RORγt in *Id2-Cre-Ert2* × *Rorc*^fl/fl^ adult mice caused an enrichment of an ex-ILC3/ILC1 RNA-seq signature, which was abrogated by the co-ablation of T-bet in *Id2-Cre-Ert2* × *Rorc*^fl/fl^ × *Tbx21*^fl/fl^ mice, as shown by the expression of ILC1 markers CD49a, NK1.1, or IFNγ [13]. This indicates that ILC3–ILC1 plasticity occurs in residential ILCs in live tissues and is not restricted to T-bet and RORγt counter-regulation in ILC progenitors.

Furthermore, induced ablation of T-bet in *Tbx21*^fl/fl^ × *Cre-Ert2* mice caused a significant loss of NKp46 expression in cLP NKp46^+^ ILC3 [20]. Similarly, GATA3 deletion in *Rorc-Cre* × *Gata3*^fl/fl^ mice also impaired NKp46 expression in SI LP NKp46^+^ ILC3 [79]. Hence, T-bet and GATA3 may collaborate in the expression of NKp46 (considered a marker); however, GATA3 has not been found to bind to *Tbx21* or promote its expression in SI LP NKp46^+^ and DN ILC3 [79]. Given that CD127 expression is increased in *Tbx21*^–/–^ cLP DN ILC3, it is surmised that T-bet counteracts the GATA3-driven expression of CD127 in intestinal ILC3 [20,72,79]. Of note, in this study, T-bet was not found to regulate RORγt or CD127 in DN ILC3 from *Rag2*-deficient mice; however, this does not preclude the possibility that T-bet regulates RORγt or CD127 via an extrinsic mechanism involving cells of the adaptive immune response [20]. From another angle, signaling through STAT1 and STAT4 can induce T-bet expression in T cells; however, RORγt expression in DN ILC3 is not altered in mice that are deficient in either one of these TF, suggesting that STAT1 and STAT4 are not necessary to induce T-bet in ILC3, because this would lead to RORγt downregulation [20,70,77].

#### TFs suppressing ILC1 differentiation

RORα has a widespread role at the transcriptional level when RORγt ablation is induced in adult *Id2-Cre-Ert2* × *Rorc*^fl/fl^ mice; this led to inhibition of an ILC1-like gene signature in SI LP NKp46^+^ ILCs [13]. The notion that RORα inhibits ILC3–ILC1 plasticity has been further supported by the low expression of RORα in SI LP, hepatic, pulmonary, and salivary gland ILC1, and by the broadly unaffected frequency of *Rora*^sg/sg^ bone marrow-derived ILC1 in the cecum upon adoptive transfer into wild-type mice [21,44,86,90]. Similarly, scRNA-seq analyses of MLN ILCs from *Salmonella* sp.-infected mice indicated that RORα inhibits a gene signature indicative of ILC1 and exILC3-ILC1, while preserving an ILC3 gene signature [86]. Overall, these data suggest that RORα and RORγt collaborate in suppressing T-bet expression, but the molecular details of this mechanism remain undefined.

**c-Musculoaponeurotic fibrosarcoma(c-Maf)** is another TF that is important in maintaining the ILC1:ILC3 balance; indeed, c-Maf is a direct repressor of *Tbx21*, leading to reduced expression of IFNγ and NKp46 in CCR6^–^ ILC3, and promotion of RORγt, IL17A, and IL22 expression in SI LP NKp46^+^ and DN ILC3, as demonstrated in *Rorc-Cre* × *Maf*^fl/fl^ compared with *Maf*^fl/fl^ control mice [34,80]. As such, c-Maf expression is high in SI LP NKp46^-^ ILC3 and low in ILC1 in adult and embryonic day (E) 18.5 mice [13,21,30,80,91]. c-Maf targeting in *Ilr7-Cre* × *Maf*^fl/fl^ or *Rorc-Cre* × *Maf*^fl/fl^ mice confirmed the role of c-Maf in promoting DN ILC3 abundance, because these mice exhibit lower frequency of this cell population compared with *Ilr7-Cre* or *Maf*^fl/fl^ mice, respectively; indeed, most studies suggest a role for c-Maf in limiting the frequency of ILC1 and NKp46^+^ ILC3 [17,34,80]. In NKp46^+^ ILC3, c-Maf was negatively regulated by IL12 and upregulated by IL7, IL23, IL15, IL1β, IL18, and Notch signaling *in vitro* [34,80]. SI LP CCR6^-^ ILC3 exhibit lower expression of c-Maf in *Tbx21*^–/–^ mice compared with wild-type mice, suggesting that c-Maf is directly or indirectly induced by T-bet; this points toward the existence of a T-bet-driven negative feedback-loop mechanism to regulate the ILC1:ILC3 balance [80].

In addition, transcriptional data from either *Il7r-Cre* × *Maf*^fl/fl^ or *Rorc-Cre* × *Maf*^fl/fl^ indicated that c-Maf also represses **B cell lymphoma 6** **(*****Bcl6*****)** expression in NKp46^+^ ILC3 because scRNA-seq analyses of these cells from *Rorc-Cre* × *Maf*^fl/fl^ and *Il7r-Cre* × *Maf*^fl/fl^ indicated lower expression of this TF compared with *Ilr7-Cre* or *Maf*^fl/fl^ mice, respectively [34,80]. Although data on the role of Bcl6 in ILC remain sparse, *Ncr1-Cre* × *Bcl6*^fl/fl^ mice exhibit lower cellularity of SI LP ILC1, while ILC3 remain unaffected, compared with *Bcl6*^fl/fl^ mice, suggesting a role for this TF in regulating the ILC1:ILC3 balance [17]. Furthermore, transcriptional analyses revealed that Bcl6 controls several target genes in ILC1 and CCR6^–^ ILC3, including the negative regulation of RORα expression [17]. Therefore, it is tempting to speculate that c-Maf-regulated Bcl6 promotes ILC1 differentiation by restraining RORα expression, although this remains to be rigorously investigated.

Another TF that influences the ILC1–ILC3 balance **is hypoxia-inducible factor 1** **(HIF1)**, which has a relevant role in the adaptation of the ILC1–ILC3 balance to hypoxia [12,37,92., 93., 94.]. Oxygen tension changes along the gastrointestinal tract with oxygen concentration being the lowest in the distal colon. Accordingly, *Rorc-Cre* × *Hif1a*^fl/fl^ mice exhibit reduced cellularity of SI LP ILC3 and a greater abundance of ILC1 relative to *Hif1a*^fl/fl^ mice; moreover, RORγt expression was diminished in SI LP ILC3, while T-bet expression was enhanced in ILC1 and ILC3, suggesting that HIF1α manipulates the ILC1:ILC3 balance in favor of ILC3 in a hypoxic environment [12]. However, T-bet expression was diminished in SI LP ILC1 and NKp46^+^ ILC3 in *Ncr1-Cre* × *Hif1a*^fl/fl^ mice relative to wild-type mice [37]. Furthermore, *Ncr1-Cre* × *Hif1a*^fl/fl^ mice exhibited a lower abundance of SI LP ILC1 and greater cellularity of SI LP NKp46^+^ ILC3 with enhanced expression of RORγt relative to wild-type mice [37]. These data indicate that HIF1α promotes T-bet expression in ILC1 and NKp46^+^ ILC3, but restrains its expression in NKp46^–^ ILC3 [12,37]. Mechanistically, HIF1α binds to the HIF1 consensus binding site in the *Tbx21* promoter region, and HIF1α expression in the ILC3 cell line MNK3 appears to be induced by hypoxia as well as by stimulation with IL1β in combination with IL23 [12,37]. This might indicate that HIF1α directly regulates T-bet expression, and this function might be elicited by both hypoxia and cytokine-mediated stimulation.

c-Maf and HIF1α appear to be direct repressors of *Tbx21* expression in ILC3, and this function supports the additional T-bet counter-regulation driven by RORα and RORγt. It is not known whether these TFs also interact in suppressing *Tbx21* expression. c-Maf is also a repressor of *Bcl6* expression, and this TF appears to collaborate with T-bet in shifting the ILC1:ILC3 balance toward ILC1.

#### ILC1 differentiation

The TF **Ikaros family transcription factor IKZF3** **(Aiolos)** may also have a role in ILC3-ILC1 plasticity; its expression in MNK3 cells represses the ILC3 lineage in conjunction with T-bet [79]. In support of this, Aiolos expression was detected in SI LP ILC1 and NKp46^+^ ILC3, while a low to high heterogenous expression profile was detected in DN ILC3 [13]. This suggested that Aiolos collaborates with T-bet and Bcl6 to promote ILC1 differentiation and limit ILC3 abundance.

It is still unknown whether Hobit-dependent ILC1 can derive from ILC3. The frequency of RORγt-fate mapper-expressing hepatic ILC1 is not altered in *Rorc-eYFP* × *Zfp683*^–/–^mice, and Hobit is barely detected in at least MLN ILC3 [14]. Hence, ILC3-ILC1 plasticity might not occur in Hobit-dependent ILC1, although more robust data are required to clarify this. In terms of T-bet-dependent ILC1, T-bet appears to promote ILC1 differentiation with assistance from Aiolos and Bcl6, but whether these TFs interact directly to suppress ILC3 differentiation remains unexplored.

## Concluding remarks

ILCs are key orchestrators of innate immune responses and, therefore, improved knowledge of the mechanisms regulating ILC differentiation and function are required to better understand ILC-mediated immunity; as such, it is important to better understand the complex TF interplay that can control ILC1 and ILC3 functions in adult mice as well as in humans [1,4., 5., 6., 7., 8., 9.] (Box 3). In particular, whether c-Maf, AHR, GATA3, HIF1, Bcl6, and Aiolos impact mature ILCs or during development requires clarification (see Outstanding questions). T-bet and Hobit are already known to be crucial for the respective maintenance of intestinal ILC1 as well as of a subset of ILC1 that are prevalent in other tissues, such as the liver [14,19]; by contrast, RORγt is required for ILC3 development, but not maintenance [13,70,73,85]. A vastly unexplored and complex area of investigation is that which assesses the potential role of post-transcriptional regulation in ILC1:ILC3 balance. Emerging reports suggest that ILC functionality can be controlled by miRNA, noncoding RNA, or RNA-binding proteins, as reviewed recently [98., 99., 100., 101., 102.]. However, in terms of TF regulation, insight is limited to the observation that noncoding RNA *IncKdm2b* can promote the expression of the TF Zfp292, which is required for intestinal ILC3 maintenance, as evidenced by the impaired SI ILC3 cellularity in *LncKdm2b*^–/–^ mice [101]. Considering that miR-155- can mediate the regulation of T-bet and c-Maf, it is tempting to speculate that the ILC1–ILC3 balance might also be crucially regulated post-transcriptionally, although this warrants robust investigation [103,104].

Box 3 **TF interplay in human ILCs**

Most human ILC1 do not express T-bet protein despite its high transcriptional expression [2]. Nevertheless, IFNγ can be induced in human tonsil NKp44^+^ ILC3 under the influence of IL12, and RORγt is induced by IL23 and IL1β in intestinal and tonsil ILC1 and NKp44^+^ ILC3 [2,28,29,31]. This indicates that the ILC1:ILC3 balance is also actively regulated in humans, but it is unknown which other TFs also have a role. However, c-Maf expression was detected at the transcriptional level in human colonic ILC3, and Aiolos expression was detected in ILC1 but not ILC3 from human gut, tonsils, and lungs [31,32,95].

A crucial role of Aiolos in human ILC3-ILC1 plasticity was further supported by the observation that stimulation of tonsil ILC3 with TGFβ in conjunction with IL23 caused RORγt downregulation and enhanced expression of T-bet and Aiolos [95]. Aiolos expression in human tonsillar ILC3 was also induced by IL1β and further influenced by IL12; IFNγ production from these ILC3 was partially abrogated in the presence of lenalidomide, a nonspecific Aiolos inhibitor [31]. Conversely, lenalidomide induced IL22 expression in human tonsillar ILC3 stimulated with IL23 and IL1β [31]. *AHR* expression was also detected in human ILC1, NKp44^–^ ILC3, and NKp44^+^ ILC3 from human tonsils and gut, but its functional role remains to be determined [28,96].

Data on HOBIT expression in human ILC1 from mucosal tissues remain sparse, but initial reports indicate that HOBIT^+^ ILC1 may exist in human tonsils and peripheral blood [41,96,97]. Initially, scRNA-seq analyses from human pediatric tonsillar ILC1 revealed *ZNF683* (encoding human HOBIT) expression, while transcripts were not detected in CCR6^+^ ILC3 or NK cells from the same tissue [96]. Peripheral blood-derived NK from patients with a haploinsufficiency for *SMAD4* had enhanced *ZNF683* expression upon stimulation with TGFβ and IL2 *in vitro* [41]. Hence, human NK cell-derived ILC1 might express HOBIT, as observed in mice.

Alt-text: Box 3

Overall, a vastly unexplored research area includes assessing the chromatin landscape of ILC precursors and mature ILCs, as well as defining the pioneer TFs that are the relevant to understand the basic TF interplay framework. TF interplays driving ILC1–ILC3 and Th1–Th17 plasticity are similar in ILC and CD4^+^ T cells; in T cells, the TFs Ets1, **Runt-related transcription factor 1 (Runx1)**, and **T cell factor 1 (TCF1)** can bind to about half of the accessible chromatin sites [105] (Box 4). Accordingly, in this scenario, Ets1 was defined as a ‘housekeeper’ TF because it broadly binds to the same chromatin sites before and after activation in T cells [105]. Runx1 binds to nearly all accessible chromatin sites in T cells and is often conjugated with activation-induced TFs, such as T-bet, while TCF1 preserves chromatin accessibility to enable its replacement by activation-induced TFs in T cells [105]. It is unknown whether these TFs have a similar role in ILCs; however, TCF1 and Runx1 are known to be essential for ILC development, and Ets1 expression has been detected in murine intestinal fetal ILC3 and mature ILC1 [21,33,34,42,73]. Furthermore, IL7-mediated STAT5 activation can control chromatin accessibility in selected genes in T cells, and IL7R is expressed in early ILC progenitors and mature ILCs, which suggests that the IL7–STAT5 axis also controls chromatin accessibility in ILCs [15,73,130]. Ideally, therapeutic TF targets to manipulate the ILC1:ILC3 balance should be identified with the prerequisite that they do not affect ILC2 function (Box 5). Once we achieve greater insight into the TF interplay in mature ILC1 and ILC3, it might be possible to advance the design of candidate pharmaceutical compounds or to apply CRISPR-Cas9 technology to ideally target TFs that are relevant for the ILC1:ILC3 balance; subsequently, it might be possible to elicit a more favorable outcome in diseases such as IBD and CRC.

Outstanding questions Are T-bet-dependent and Hobit-dependent ILC1 the same set of cells, or are Hobit-expressing ILC1 fully derived from NK cells?

Does plasticity exist between T-bet-dependent ILC1 and Hobit-expressing ILC1?

Is T-bet redundant for human ILC1 functionality?

Which TFs are crucial for ILC3 maintenance? Although otherwise expected, RORγt and RORα are not essential for the postdevelopmental maintenance of ILC3. This suggests that the TF interplay supporting mature ILC3 remains unknown.

Do Aiolos and Bcl6 directly regulate RORγt expression? Does RORα directly regulate T-bet expression? Are further TFs involved in ILC3–ILC1 plasticity?

Do GATA3 and Ahr promote ILC3–ILC1 plasticity or ILC maintenance?

Which pioneer TFs are relevant for ILCs? Are Runx1, Runx3, TCF1, Ets1, and GATA3 pioneer TFs in ILCs?

From which developmental stage do c-Maf, RORα, HIF1, Aiolos, and Bcl6 have a role in ILC functionality? Do they have a developmental or post-developmental role in ILCs?

Which TFs control metabolic fitness of ILC?

Which TFs promote IL7R expression in ILC in addition to GATA3 and oppose T-bet-mediated inhibition of IL7R expression?

Does post-transcriptional regulation control the balance between ILC1 and ILC3?

Alt-text: Outstanding questions

Box 4 **TF interplay in CD4+ T cells and associations with ILCs**

Similar to ILC1, T-bet has a prominent role in driving the differentiation of Th1 cells and *Ifng* (encoding IFNγ) expression and *Cd127* (encoding CD127) repression in mouse CD4^+^ T cells [60,106]. However, there are marked differences in epigenomes at the *Tbx21* locus [77]. The *Tbx21-CNS3* site is not accessible in Th1 cells, while it is relevant for T-bet expression in hepatic ILC1 [77]. However, STAT4 binding to *Tbx21-CNS3* is essential for inducing T-bet expression in Th1, but STAT-binding motifs at this site are dispensable for hepatic ILC1 [77]. T-bet interacts with GATA3 when driving the expression of T-bet-specific genes, and this is sufficient to distribute GATA3 away from Th2-specific genes [107., 108., 109.]. GATA3 shares binding sites with T-bet and directly represses IFNγ expression via the recruitment of polycomb repressive complex-2, unless it is conjugated to T-bet [110,111]. The ‘amplifying’ TFs Runx1 and Runx3 are important for Th1 and Th17 differentiation [108,112]. T-bet:Runx1 heterodimers prevent Runx1-driven expression of *Rorc* (encoding RORγt), and both T-bet:Runx1 and T-bet:Runx3 complexes promote IFNγ expression in CD4^+^ T cells [108,112]. RORγt has a key role in the development of Th17, and RORγt:Runx1 is necessary to drive IL17A and IL22 expression in mouse models [113., 114., 115.]. Similar to ILC3, RORα has a synergistic role with RORγt in driving Th17 responses; this was demonstrated by the reduced production of IL17A in human CD4^+^ T cells *in vitro* in the presence of either RORγt or RORα short interfering (si)RNA. Moreover, there was greater intestinal Th17 cell abundance in *Rag1*^–/–^ mice upon reconstitution with wild-type bone marrow compared with the respective source of *Rorc*^–/–^ × *Rora*^sg/sg^ mice [116,117]. In addition, AHR was shown to promote IL22 production in Th17 cells, given that murine *Ahr*^–/–^ Th17 cells could not produce this cytokine *in vitro* and in an EAE model [88]. Furthermore, oral administration of the AHR agonist precursor indole-3-pyruvic acid in mice also suppressed IFNγ expression in CD4^+^ CD8αα^+^ IEL lymphocytes [88].

However, there are also differences in T cells in terms of ILC3 and ILC1, because, unlike what has been reported in ILC1 and ILC3, c-Maf represses RORγt and IL22 in Th17, and Aiolos promotes Th17 differentiation via IL2 silencing [118,119]. Aiolos also promotes *Bcl6* transcription in follicular helper T cells, a mechanism not explored in ILC [120]. In contrast to ILC, Bcl6 plays a key role in the development and maintenance of T_FH_ via repression of Th1, Th17, and Th2 differentiation [121,122].

In addition to Hobit-dependent ILC1, HOBIT is also expressed in cytotoxic T-bet^+^ CD4^+^ T cells from human peripheral blood and has been associated with the formation of tissue-resident memory CD4^+^ T cells in mice [43,123,124]. In murine NKT cells, Hobit restrains IFNγ and promotes granzyme B production [125]. While the role of **B lymphocyte-induced maturation protein 1** **(Blimp1)** expression in ILC is unknown, it is key for granzyme B production and for the IL4-induced expression of IL10 in Th1 cells [126., 127., 128., 129.].

Alt-text: Box 4

Box 5 **TF interplay in ILC2**

Many of the TFs regulating the ILC1:ILC3 balance have also been detected in ILC2. This is of key relevance because targeting these TFs to manipulate the ILC1:ILC3 balance might also affect the activity of ILC2 [2,3]. For instance, choosing RORα as a target might result in significant ablation of ILC2, as demonstrated by the loss of intestinal and pulmonary ILC2 in *Rora*^sg/sg^ mice [131]. Alternatively, targeting GATA3 might also result in the loss of ILC2, as demonstrated by the induced ablation of SI LP ILC2 in *Cre-Ert2* × *Gata3*^fl/fl^ mice [22,79]. Despite the key role of GATA3 in mature ILC2, a role for the GATA3–AHR axis for ILC3 and ILC1 does not appear to be relevant for ILC2. In fact, *Ahr*^–/–^ mice have greater abundance of SI LP ILC2 compared with wild-type mice, and overexpression of AHR in *Ahr*^dCAIR/+^ mice results in diminished cellularity of cLP ILC2 relative to wild-type mice [15]. Furthermore, Aiolos enhances GATA3 and RORα expression in cLP ILC2, as demonstrated by targeting *Ikzf3* (encoding Aiolos) using Ikzf3-shRNA *in vitro* [132].

Plasticity also occurs in murine and human ILC2, leading to expression of T-bet or RORγt in these cells, as recently reviewed [3]. Murine pulmonary ILC2 express IFNγ and T-bet upon stimulation with IL12 and IL18 *in vitro* [90]. The key role of IL12 in driving IFNγ and T-bet expression was also observed in human peripheral blood ILC2 *in vitro* [90,133., 134., 135., 136.]. Moreover, preliminary data of fate-mapper and reporter mouse models for T-bet demonstrated that ~5% of intestinal ILC2 express T-bet [137]. In MLN ILC2, GATA3 appears to bind to an intron in *Tbx21*, perhaps resulting from a direct interaction between T-bet and GATA3 as in CD4^+^ T cells, and perhaps due to the redistribution of GATA3 away from GATA3-target genes [79,138]. This notion is further supported by the greater abundance of intestinal ILC2 in *Tbx21*^–/–^ mice relative to wild-type mice, which might indicate that T-bet restrains ILC2 differentiation intrinsically in ILC [72]. Alternatively, although conjectural, T-bet deficiency might affect ILC2 abundance extrinsically. Likewise, although c-Maf is a direct repressor of T-bet, the ablation of c-Maf in *Il7r-Cre* × *Maf*^fl/fl^ causes a greater abundance of intestinal ILC2 relative to *Maf*^fl/fl^ or *Il7r-Cre* mice, suggesting that c-Maf inhibits ILC2 via an extrinsic mechanism [17,34]. Hobit expression has not been explored in ILC2 in mucosal tissues and was not detected in MLN ILC2 [14]. RORγt expression in murine pulmonary KLRG1^low^ ILC2 is induced by Notch signaling, as demonstrated by an *in vitro* co-culture with OP9-DL1 stroma compared with OP9 cells [139]. RORγt expression was also observed in human peripheral blood ILC2 when stimulated with TGFβ in conjunction with IL1β and IL23 *in vitro* [140].

Alt-text: Box 5

## Glossary

**Aryl hydrocarbon receptor (AHR)**: TF promoting Th17 and ILC3 functionality.
**Assay for transposase-accessible chromatin using sequencing (ATAC-seq)**: molecular technique allowing genome-wide detection of chromatin accessibility.
**B cell lymphoma 6 (BCL6)**: TF driving follicular T helper cell differentiation; expressed in ILCs.
**B lymphocyte-induced maturation protein-1 (Blimp1)**: TF expressed in subsets of T cells and ILCs.
**c-Musculoaponeurotic fibrosarcoma (c-Maf)**: TF with high expression in ILC3.
**Cre-Ert2**: fusion protein comprising Cre recombinase fused to a triple mutant form of the human estrogen receptor. Entry of Cre-Ert2 into the nucleus is regulated by tamoxifen binding to Ert2.
**Dextran sulfate sodium**: a sulfated polysaccharide used in mouse models to induce colitis via its toxicity to the colonic epithelium.
**ex-ILC3**: ILC1 derived from an ILC3 precursor.
**Follicular T helper cells (T**_**FH**_**)**: CD4^+^ T cell subset resident in secondary lymphoid organs; key role in promoting the selection and survival of B cells.
**GATA-binding protein 3 (GATA3)**: key TF driving Th2 differentiation, ILC development, and ILC2 maintenance.
**Homolog of Blimp-1 in T cells (Hobit)**: TF expressed in subsets of ILC1 and T cells.
**Hypoxia-inducible factor 1 (HIF1)**: hypoxia-inducible TF expressed in CD4^+^ T cells and ILC subsets; comprises subunits HIF1α and HIFβ.
**Ikaros family transcription factor IKZF3 (Aiolos)**: TF expressed in T cells and ILCs.
**Intraepithelial lymphocytes (IELs)**: lymphocytes found in the epithelial layer of mucosal tissue.
**Lamina propria (LP)**: layer of connective tissue underneath the epithelium in the intestine.
**Mesenteric lymph node**: gut-draining secondary lymphoid tissue.
**OP9-DL1**: stromal cell line expressing the Notch ligand delta-like 1 (DL-1) used for T cell and ILC *in vitro* culture systems.
**RAR-related orphan nuclear receptor γt (RORγt)**: TF expressed in Th17 and ILC3.
**Regulatory T cells (Treg)**: subpopulation of T cells with an immunosuppressive function.
**Retinoic acid receptor-related orphan receptor α (RORα)**: TF highly expressed in ILC3 and ILC2.
**Runt-related transcription factor (RUNX)**: Runx1 and Runx3 are key TFs expressed in T cells and ILCs.
**T cell factor 1 (TCF1)**: key TF in T cells and ILCs.
**T-box expressed in T cells (T-bet)**: TF expressed in Th1, CD8^+^ T cells, ILC1, NKp46^+^, ILC3, and NK cells.
**Trajectory analysis**: computational technique used in RNA-seq analyses to determine the pattern of a dynamic process experienced by cells and then to arrange cells based on their progression through the process.
